# The Intersection of Age and Influenza Severity: Utility of Ferrets for Dissecting the Age-Dependent Immune Responses and Relevance to Age-Specific Vaccine Development

**DOI:** 10.3390/v13040678

**Published:** 2021-04-15

**Authors:** Melissa Rioux, Magen E. Francis, Cynthia L. Swan, Anni Ge, Andrea Kroeker, Alyson A. Kelvin

**Affiliations:** 1 Department of Microbiology and Immunology, Faculty of Medicine, Dalhousie University, Halifax, NS B3H4R2, Canada; melissa.rioux@dal.ca (M.R.); an848259@dal.ca (A.G.); 2 Vaccine and Infectious Disease Organization (VIDO), University of Saskatchewan, Saskatoon, SK S7N5E3, Canada; m.francis@usask.ca (M.E.F.); cynthia.swan@usask.ca (C.L.S.); andrea.kroeker@usask.ca (A.K.); 3 Department of Pediatrics, Division of Infectious Disease, Faculty of Medicine, Dalhousie University, Halifax, NS B3K6R8, Canada; 4 The Canadian Center for Vaccinology (IWK Health Centre, Dalhousie University and the Nova Scotia Health Authority), Halifax, NS B3K6R8, Canada; 5 Department of Biochemistry, College of Medicine, University of Saskatchewan, 107 Wiggins Road, Saskatoon, SK S7N5E5, Canada

**Keywords:** ferret, preclinical model, influenza, respiratory viruses, age-related disease, risk-factors, host response, immune response, correlate of disease, correlate of protection

## Abstract

Many factors impact the host response to influenza virus infection and vaccination. Ferrets have been an indispensable reagent for influenza virus research for almost one hundred years. One of the most significant and well-known factors affecting human disease after infection is host age. Another significant factor is the virus, as strain-specific disease severity is well known. Studying age-related impacts on viral infection and vaccination outcomes requires an animal model that reflects both the physiological and immunological changes that occur with human aging, and sensitivity to differentially virulent influenza viruses. The ferret is uniquely susceptible to a plethora of influenza viruses impacting humans and has proven extremely useful in studying the clinical and immunological pictures of influenza virus infection. Moreover, ferrets developmentally have several of the age-related physiological changes that occur in humans throughout infancy, adulthood, old age, and pregnancy. In this review, we discuss ferret susceptibility to influenza viruses, summarize previous influenza studies using ferrets as models of age, and finally, highlight the application of ferret age models in the pursuit of prophylactic and therapeutic agents to address age-related influenza disease severity.

## 1. Introduction

### 1.1. The Impact of Host Factors such as Age

Influenza is a public health burden typically causing yearly epidemics, resulting in approximately 1 billion cases worldwide and as many as 650,000 deaths annually [1]. Influenza viruses mainly target the respiratory tract, and disease severity can range from mild to severe disease resulting in death. Symptoms of influenza include coughing, sneezing, and fever in mild cases, and bronchitis, alveolitis, and pneumonia leading to DAD (diffuse alveolar damage) and ARDS (acute respiratory disease syndrome) in those severely affected. Certain host factors are known to be associated with severe disease and poor outcomes [2]. These risk factors include diabetes, pregnancy, obesity, and age. It is well known that the extreme age groups, the very young and the very old, are more vulnerable to severe influenza. Approximately 90% of influenza-related deaths, and 50–70% of influenza-related hospitalizations, occur in individuals above 65 years and older [3]. Despite the broad understanding of age as a risk factor for severe influenza, how age influences disease and the immune or physiological mechanisms regulating pathology remains a significant research priority.

### 1.2. The Influenza Virus Family

The influenza virus family (Orthomyxoviridae) of segmented negative sense RNA viruses is grouped into four antigenically distinct virus types: A, B, C, and D [4,5]. Influenza A and B viruses, each with its own subtypes and lineages, are the main seasonal, epidemic-causing viruses circulating in humans. These virus types consist of eight single-stranded RNA segments that can express up to 12 proteins: polymerase acidic (PA/PA-X), polymerase basic 1 (PB1/PB1-F2), polymerase basic 2 (PB2), hemagglutinin (HA), nucleoprotein (NP), neuraminidase (NA), matrix protein 1 and 2 (M1/M2), non-structural protein 1 (NS1), and nuclear export protein (NEP/NS2) [6]. The HA, composed of an immunodominant globular head domain attached to a more conserved stalk domain, is responsible for host cell entry. For entry, the HA molecule binds to sialic acids, *α*2,3- or *α*2,6-linkages to galactose, which line the respiratory tract [7]. The HA globular head is variable and frequently mutated since it is a target for antibody-dependent virus neutralization. Influenza A viruses are identified by their surface proteins HA and NA, where there are 18 HA and 11 NA proteins that combine to designate subtypes, such as H1N1 and H3N2 [6]. The animal reservoir of all influenza A viruses is aquatic birds. This virus type of non-human origin has the capacity to move between animal and human hosts, permitting a large degree of disease and destruction, although such spillover events are rare. Influenza B virus has a more limited host range and has only been shown to infect humans, seals, and experimental animals such as ferrets [8,9]. Type B viruses are not categorized by subtypes but instead are defined by two lineages (B/Victoria and B/Yamagata lineage), each of which is also comprised of antigenically evolving strains [10].

Influenza viruses are prone to genetic variability through two mutational mechanisms: antigenic shift and antigenic drift [11]. Antigenic drift is the accumulation and retention of viral mutations during replication, whereas antigenic shift is the swapping of viral genomic segments between different viruses as they infect the same cell at the same time [11]. Antigenic shift can yield novel influenza viruses with pandemic potential and cause increased morbidity in populations that have not previously been exposed [1,12,13]. Although antigenic drift only yields point mutations, it causes extensive antigenic diversity and is responsible for recurring seasonal epidemics [6].

### 1.3. The Importance of Age, the Dynamic Host Factor

The host’s immune response during influenza virus infection dictates disease severity. Age is known to play a major role in regulating the key profiles of host immunity [14,15,16], but the mechanisms of age-dependent immune regulation of viral infection have not been fully elucidated.

The early stages of influenza virus infection are defined by the antiviral response and innate immunity [17]. Evidence suggests that infants and young children have decreased antiviral responses due to lower signaling of receptors, which bind pathogen-associated molecular patterns (PAMPs), such as viral nucleic acids [14]. These receptors include the toll-like receptors, which are referred to as pattern recognition receptors (PRRs). Conversely, the older immune system has been associated with a condition referred to as “inflamma-aging”, which is a low, constitutive level of inflammation present in aged individuals [16]. Both decreased PRR activity and inflamma-aging have been connected to poor antiviral responses in the extreme age groups. Subsequently, following a typical antiviral and innate immune response, adaptive cellular (cytotoxic CD8^+^ T cells) and humoral responses (antibody-producing B cells) are activated to establish pathogen-specific responses and immune memory [18]. As with the initial responses to viral infection, the adaptive responses also deviate from those of average adults. Infants have a well-known Th2 bias of the adaptive immune system, while older individuals experience immunosenescence and T cell exhaustion [14,19,20]. Additionally, the interplay between memory B cell (MBC) subsets, specifically the CD27^dull^ and CD27^bright^ MBCs, differs across age groups [21]. The MBCs of young children are predominantly of the CD27^dull^ subset and allow for rapid, broadly reactive antibody production at infection, while older individuals possess highly specific CD27^bright^ MBCs that are incapable of adaptation to new antigens [22]. These MBC subsets are hypothesized to impact the host capacity to generate a humoral immune response to novel pathogens, and thus susceptibility to infections, such as SARS-CoV-2 [21,22]. Importantly, the mechanisms regulating these processes and how age-related immune conditions skew the host–virus interaction are poorly understood.

### 1.4. How to Study Age-Related Influenza

The use of animal models has been a key tool in elucidating both the pathogenic characteristics of epidemic and emerging influenza viruses, and aspects of the host, which influence disease severity. The ferret (*Mustela putorius furo*) has become a primary animal model for studying influenza viruses and evaluating vaccines and therapeutics. This is in part due to similarities in ferret and human lung physiology, including the distribution of sialic acid moieties throughout lung tissues and thus influenza virus tropism throughout the respiratory tract [13]. Physiologically, ferrets undergo similar immune maturation, respiratory development, and aging as humans, allowing age-related investigation as our group and others have done [14,23,24,25,26,27,28,29,30,31]. Additionally, ferrets are susceptible to influenza virus infection through both aerosol and non-aerosol routes. Clinical symptoms that manifest in the ferret have been shown to be strain-specific, and symptoms more closely mimic symptoms seen in humans compared to mouse or guinea pig models [13]. This has allowed the analysis of specific disease-causing characteristics of individual influenza virus strains [32]. Ferrets are also susceptible to the same zoonotic influenza strains that can infect humans, demonstrating that the ferret is an ideal model to study emerging influenza virus strains with pandemic potential [1,13]. In this review, we discuss the utility of ferrets to dissect not only the clinical aspects of strain-specific influenza virus infection, but also how the ferret model can be used to elucidate the age-related influence on viral-induced immune responses and infection outcomes.

## 2. Ferrets Are Uniquely Susceptible to Different Influenza Viruses

A prerequisite to accurate modeling of host age-related impacts on influenza virus infection is sensitivity to influenza viruses. Ferrets are naturally susceptible to influenza virus types A and B, which has led to focused investigations in adult ferrets [33]. Ferrets have been an invaluable model of human influenza virus infection for several decades, as they display clinical profiles that mirror human symptoms in response to influenza virus infection (reviewed here [34]). Like humans, ferrets can develop fever, sneezing, nasal secretions, lymphopenia, lethargy, and weight loss, allowing the investigation of clinical manifestations and disease severity after infection. Additionally, since ferrets are susceptible to viral infection in the upper respiratory tract and the lower, ferrets are able to transmit the virus through direct contact and by aerosols through the acts of coughing, sneezing, or breathing [13]. This feature of the ferret allows the modeling of transmission dynamics and the aerosol transmission potential of new and emerging influenza viruses and other viruses such as the current pandemic virus SARS-CoV-2 (severe acute coronavirus 2) [35,36,37]. Through years of research, ferrets have clearly shown that the clinical course of infection, as in humans, is dependent on both host factors such as obesity, pregnancy, immune status, host age, and the specific strain [33,38]. Moreover, ferrets have proven useful for identifying correlates of disease and protection that are strain-specific [39]. As we recognize that disease severity is dependent on both host factors and the infecting virus strain, we start this review by examining the historical role ferrets have played in influenza research and how influenza studies are carried out using the ferret. This will provide a foundation at which to compare age-related analysis in the upcoming sections.

### 2.1. Clinical Disease and Disease Dissection in Ferrets Following Influenza Virus Infection

Ferrets have historically been connected to influenza [40]. In the 1930s, English scientists Wilson Smith, Christopher H. Andrewes, and Patrick P. Laidlaw inoculated ferrets using human clinical throat and lung samples from people experiencing influenza during the 1933 epidemic [41]. After two days, the ferrets experienced observable clinical disease. It was the manifestation of human influenza-like disease that allowed researchers to eventually determine that the influenza virus was the etiological agent of influenza, leading to a culturing method of the virus (propagation of the viral pathogen in ferrets) and allowing eventual development of a vaccine. We now recognize the ferret for its unique ability to manifest the human-like symptoms of influenza virus infection, and infection with other respiratory viruses. Clinical signs of influenza virus infection in ferrets include fever, weight loss, inactivity, inappetence, sneezing, coughing, and nasal discharge [42]. Importantly, as we highlight later in this review, these signs differ in magnitude depending on the infecting viral strain and offer a tool for examining strain-specific severity and therapeutic evaluation, and disease skewing by the age of the host.

As ferrets are susceptible to influenza virus strains from species such as humans, avian, equine, and swine, clinical assessment in this model has allowed a more quantitative understanding of disease caused by specific influenza types, subtypes, lineages, and strains. Study methodology and rigor are important for the accurate quantification of clinical disease [43], although applied methods for measuring disease manifestation in ferrets are not universal. In our experience, animals should be monitored at the same time each day for consistency, and to avoid confusion with daily circadian rhythms changes. Temperature can be measured by an implanted temperature chip or by use of a rectal thermometer. Both temperature and weight should be expressed in terms of percentage of original values, which is determined over a baseline of a few days to account for day-to-day fluctuation. Activity level can be determined by a scoring system [44,45] as follows: 0, alert and playful; 0.5, alert but playful only when stimulated; 1, alert but not playful when stimulated; and 2, neither alert nor playful when stimulated. A relative inactivity index is calculated using the formula:

Σ_(day 1 to day 14)_[score + 1]*_n_*/Σ_(day 1 to day 14)_*n*

where *n* equals the total number of observations. A value of 1 is added to each observation unit score so that a score of 0 can be divided by a denominator, resulting in an index value of 1.0 as the minimum value [32]. When observing the clinical features, the nose and anus of each animal should be examined daily for the presence and color of wet or dry discharge from the nose or wet or dry fecal matter from the anus. Nasal discharge can include crusty nose, mucous, and transparent exudates/fluids. While performing clinical assessments, sneezing and coughing can be observed over a consistent time frame each day. The sneezing and coughing scores can be calculated from the total animals found sneezing over the total number of animals [32]. More sophisticated methods have now been developed, which allow continual monitoring of animal clinical signs by telemetry devices or surveillance video [43]. Neurological abnormalities should also be assessed, especially when considering infection with influenza viruses that may lead to extrapulmonary tropism. Together, we and others employ parameters which indicate the development of mild, moderate, and severe disease [25,32,46]. For example, mild disease is generally characterized by less than 5% of the original weight being lost, and temperature increases less than 2% for a single day. Conversely, severe disease parameters include the loss of greater than 15% weight and prolonged fever over several days typically exceeding 40 °C or 103% of the original temperature.

### 2.2. Using Ferrets to Establish Correlates of Pathogenesis and Protections: Immune Responses to Influenza Virus Infection in Ferrets

The similarity of the immunological responses between the ferret model and humans designates the ferret as an attractive model for identifying correlates of severe disease and immune-induced protection [47]. However, it is generally considered that reagents are limited for immunological dissection in the ferret model (Table 1). Significant strides have been made in recent years to expand the ferret immunological toolbox, including both the sequence of the ferret transcriptome, infectome, glycome, and B cell receptor repertoire, and T follicular helper cell identification [38,46,48,49,50,51]. Classically, ferret humoral responses were evaluated after virus infection and vaccination by antibody quantification and function through hemagglutinin inhibition (HI) assays and virus neutralization assays, respectively. With increased immunological studies, we now have more than 30 antibodies validated for flow cytometry or immunohistochemistry for targets such as STAT3, CD20, and TNF [23,24,38] (Table 1). Additionally, for larger scale profiling, we have validated ferret PCR primer sets for almost 100 immune gene targets [23,24,50,52,53]. Although immune subset refinement remains a challenge, OMICS technologies have increased the capacity of immune resolution that can be applied to pathogenesis influenza studies, and determining immune mechanisms of protection in vaccine studies.

## 3. Influenza in the Infant and Newly Weaned Ferret

Immunologically speaking, early life is marked by significant changes that occur quite quickly with respect to the total human lifespan. Additionally, human infants and neonatal ferrets are born with lungs that will continue to develop after birth. Prior to childhood, infancy can be marked by three stages: neonatal, infant, and toddler. The highest mortality due to influenza virus infection occurs during the neonatal stage, which then begins to decline with the transition into infancy and toddler ages [14]. Early life development is marked by immune and respiratory function maturation, possibly contributing to the decline of influenza-associated mortality. Maternal immunoglobulin G (IgG) is high in full-term neonates and decreases rapidly over the first few months of life, highlighting the dependency of the neonate on maternal immunity. At birth, the immune system is biased toward T helper cell type 2 responses, but maturation over the first 2–3 years of life is characterized by increased T helper cell type 1 responses and increases in the infants’ ability to produce antibodies, thereby altering host responses to pathogens. Infants also regulate immunomodulatory factors differently from adults. As well, infants have lower levels of antigen presenting cells. Interestingly, influenza virus infection during infancy is now understood to imprint the immune system, thereby setting the immunological tone for influenza outcomes for the remainder of one’s life. Understanding how the early-life host responses vary from those of the adult, and how these responses are connected to disease outcome after influenza virus infection remains a research priority, especially considering influenza vaccine development. It is well known that vaccine responses are blunted in infants and early childhood, further pressing the need for effective influenza vaccines specific for early life. Ferrets have been employed to understand the pathogenesis of influenza virus infection during early life. The establishment of early-life models of influenza pathogenesis allows the evaluation of next-generation vaccines designed for vulnerable young age groups.

### 3.1. Influenza Disease in Infant Ferrets

**Figure 1:**
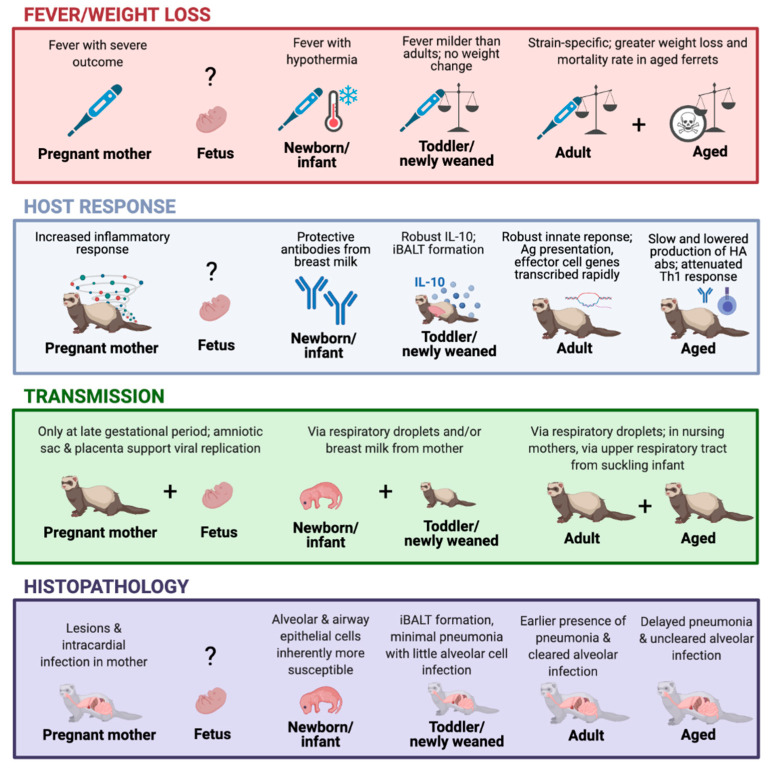
The ferret model timeline in response to influenza virus infection. The graph depicts the viral infection-induced illness, host response, transmission, and histopathology outcomes at different stages of the ferret lifespan, including pregnancy [24,29,75], fetus [29,31,76], newborn/infant [26,27,28,30,72,73,74], toddler/newly weaned [23,25], adults [24,50], and aged animals [25,77,78]. Created with BioRender.com.

Infants are classically known to be highly susceptible to severe disease, leading to hospitalization following influenza infection. As young ferrets are still undergoing maturation of the immune and respiratory systems while remaining susceptible to infection with human influenza viral isolates, the neonatal ferret is an essential (and perhaps the only) preclinical model capable of dissecting infant immune responses during infection of specific influenza strains. Early studies of newborn ferrets show a greater susceptibility to infection in alveolar cells compared to older animals [26] (Figure 1). Coates et al. utilized the recombinant influenza virus A/PR/8/34-A/England/939/69 clone 7a (H3N2) to inoculate newborn (1 day old) and suckling animals (15 days old) intranasally at 10^0.9^ EBID_50_. An additional group of suckling ferrets was inoculated at 10^6^ EBID_50_ [26], a dose previously shown to be non-fatal in adult ferrets [64]. Viral load was consistently higher in the lower respiratory tract compared to the upper respiratory tract of newborn ferrets inoculated at 10^0.9^ EBID_50_. By day 9 post-infection, all newborn ferrets still showed virus in the lungs and nasal turbinates, and ultimately succumbed to infection. While similar titers were seen in the lower respiratory tract of suckling ferrets inoculated at 10^0.9^ EBID_50_ on the first 7 days post-inoculation, on days 7 and 8 there was a significant decrease in the viral load in this group compared to newborn animals, with no virus detected on day 9 [26]. In the suckling ferrets inoculated at a higher dose, the virus was still able to be eliminated from the respiratory tissues by day 9 and only 3 of the 25 animals assessed succumbed to illness during the study. When observing the location of infected cells in the respiratory tract via fluorescent staining of the virus, suckling ferrets, regardless of low or high dose, showed peak presence of virus on day 3, with a gradual decrease over the remaining days. In addition, only the bronchial epithelium was affected. In contrast, newborn ferrets infected at a lower dose not only showed a consistent amount of virus throughout the time course, but also had virus present in the bronchiole and the alveolar region [26]. Husseini et al. showed that neonatal ferrets mimic neonatal humans in that they are also predisposed to secondary bacterial infection that often result in death [27]. This group utilized the parental strain of clone 7a, A/PR/8/34, to infect neonatal ferrets. Infected animals were treated with antibiotics post-inoculation to reduce the possibility of secondary bacterial infection. When neonates infected with A/PR/8/34 also received antibiotics, death rates dropped by 39% in the non-antibiotic treated animals. While viral pneumonia can be severe in these animals, it is hypothesized that it is the obstruction of the airway due to the underdeveloped respiratory system that causes mortality [27]. Collie et al. assessed influenza virus infection in newborns by infecting neonatal ferrets with A/PR/8/34-A/England/939/69 clone 7a, an H3N2 virus, at various doses [28]. Even at low doses, these infections led to mortality in the newborn ferret. These animals had severe lesions in the upper respiratory tract. These studies also concluded that obstruction of airways and esophageal passages were the cause of death, suggesting that difficulty eating contributed to mortality in addition to respiratory pathology [28]. Our 2015 study demonstrated the effect of infection with 2009 pandemic H1N1 in suckling infants [24]. Dual transmission was demonstrated after the A/California/07/2009 virus infection, which caused significant respiratory tract disease in mothers and infants, where all infants succumbed to disease [24]. Unexpectedly, we also found that influenza virus can be found in the breast milk and mammary tissue of mothers whose suckling infants have been infected, suggesting an additional mode of virus transmission in the mother-infant-dyad [24]. Data from this study suggested that the virus infected glandular epithelial cells in the mammary tissue. Four-week-old suckling ferrets were infected intranasally, leading to a fever of 104 °C by day 2 post-inoculation, followed by hypothermia by day 6 post-inoculation (Figure 1). All inoculated animals reached humane endpoints or died by day 8 post-inoculation. These animals began showing viral shedding by day 1 post-inoculation. When mothers of four-week-old suckling infants were intranasally inoculated, they showed viral shedding in the nasal washes by day 3 post-maternal inoculation. Infants began losing weight on day 3 post-inoculation and died by day 11. Infant ferrets became hypothermic as they reached mortality. Virus was detected in the lungs on days 5 and 8, leading to severe pathology noted on day 7 post-maternal inoculation. Infants showed dense leukocyte infiltration and destruction of the alveoli. Viral RNA was found not only in the respiratory tract of suckling infants, but also in the feces, supporting that both respiratory and gastrointestinal tract are possible routes of infection for the infant. When the mammary gland of the nursing mother was infected directly, this too led to severe outcomes for infant ferrets, with only 30% survival by day 7 post-inoculation [24]. This mode of transmission was not identified prior to this study and provided insight into the immune regulation within the mammary gland related to pathogen-specific antibody production [24].

With increased understanding of influenza virus diversity and the history of pandemic and epidemic influenza, the importance of influenza imprinting on disease outcomes and vaccine design has been recognized by us and others [6,14,65,66,67,68]. Influenza imprinting is defined as the first influenza virus infection during infancy. Specifically, evidence has indicated that this first infection during early life establishes life-long antibodies specific to the first virus [69,70]. It has been hypothesized that the infant immune system may facilitate a broader immune response than those of the more immunologically mature ages [14]. In our discussion of age and ferret models for influenza, it is important that we mention that ferrets have been proposed as a model for understanding influenza virus imprinting of the immune system [67,71]. With increased need to understand influenza imprinting for pathogenesis modeling and universal vaccine design, the infant ferret model may be the key to determining the mechanisms of strain-specific imprinting in infants and the subsequent influence this established immune memory has on future infection and vaccination events.

With respect to influenza virus infection and vaccination outcomes, several studies have demonstrated the importance of breastfeeding in the infant ferret. Infants gain passive immunity from mothers through breast milk, receiving influenza virus-specific IgG antibodies [26,30,72,73,74] (Figure 1). In a 1984 study investigating protection afforded from mother’s breastmilk against severe influenza, 1-day-old newborn ferrets were either allowed to suckle from their mother or were fed an artificial diet prior to influenza virus inoculation [26]. The study found that while suckling neonates were well-protected against challenge, those infants that were not fed mother’s milk ultimately succumbed to influenza virus infection [26]. Specifically, influenza virus infection in non-suckling neonates progressed quickly, with both alveolar and bronchial cells being infected. In contrast, disease in suckling neonates was less severe and minimally impacted alveolar tissue. Another study by Jakeman and colleagues showed that vaccinated mother ferrets pass on protective antibodies to their suckling infants. Mothers who received an attenuated influenza vaccine had infants with higher levels of anti-hemagglutinin antibodies. However, infants of mothers who received a live vaccine had better protection, leading the authors to conclude that influenza viral proteins apart from HA played an important role in passive protection in infants [30]. Contradictory to passive immune mechanisms of protection suggested in the studies above, another study investigating passive protection using the ferret model revealed that while influenza virus IgA and IgM antibodies are both present in the milk from of vaccinated mothers, these antibodies do not cross the neonatal gut epithelial border and therefore do not contribute to protection [72].

### 3.2. The Newly Weaned Are Not Infants or Neonates—Differential Disease in the Toddler Age Group of Ferrets

We extended the study of young ferrets by establishing a newly weaned ferret model to assess outcomes of the 2009 H1N1 pandemic [79]. The progression out of infancy is marked by a significant lowering of susceptibility to severe influenza [80]. Although the toddler age group may still have higher susceptibility than healthy adults, the highly vulnerable population are those prior to 6 months, followed by those younger than 2 years of age. Immunologically, toddlers are quite different from infants as they have a more balanced Th1/Th2 immune response and now typically consume solid foods, thereby changing their antigen exposure and gut-immune development, so they rely less on immunity from their mothers [14]. Considering the difference in developmental stages between infant and newly weaned animals, we designed a study to investigate the clinical disease, outcome, and immunological responses of recently weaned ferrets infected with the A/Mexico/4108/2009 strain of the pandemic 2009 H1N1 lineage, which was shown to cause 100% mortality in infant ferrets and moderate/severe disease in adult ferrets [23,24,32]. Our study, led by Huang, found that newly weaned animals had a relatively mild response to infection, characterized by a mild dip in weight that quickly returned to their age-matched uninfected weight trajectory [23] (Figure 1). A milder fever was also noted when compared to infected adult ferrets. These mild responses were attributed to, in part, a more robust IL-10 response that was not seen in adult animals. Little interstitial pneumonia or bronchopneumonia were present in the lungs of newly weaned animals, which has been reported in other studies [23,25]. Importantly, structures similar to iBALT (inducible bronchus-associated lymphoid tissue) formed in the lung that were not present in adult ferrets. These were associated with an immune and cytokine profile consistent with iBALT, including increased levels of chemokines CCL19 and CXCL13, and increased leukocytes in the lung [23]. iBALT is a tertiary lymphoid structure in the lungs and bronchus that is induced upon damage to the lung and is typically associated with infection. iBALT serves as an area for T and B cell stimulation as a result of antigen encounter [81]. Taken together, the results of this study agreed with preclinical studies that protective iBALT immune responses emerge with respiratory insult in younger animals [81,82]. From this, it was hypothesized that iBALT, which was not observed in infected infant or adult ferrets, may play a protective role in newly weaned influenza virus infection and clinical disease outcome [23,24].

## 4. The Gold Standard of Influenza Models: The Adult Ferret and Its Use to Dissect Strain-Specific Disease

Ferrets are the gold standard for modeling influenza virus infection and for testing vaccines and therapeutics. The most widely used model is the adult model aged 8 months to 1 year. This model is the most defined, but outcomes do vary based on the variable influenza viruses used in specific studies as we review here. Seasonal influenza viruses are continually drifting and therefore understanding previous seasonal viruses and current seasonal viruses is essential.

### 4.1. Seasonal Influenza in Adult Ferrets

Previously published studies using the ferret model have generally found that seasonal influenza A strains H1N1 (1977 lineage) and H3N2, and influenza B strains, cause the mildest clinical outcome; pandemic influenza H1N1 (2009 and 1918) strains lead to a moderate outcome; and finally, the most severe strains causing lethality have been avian influenza strains of the subtype H5N1 [83,84] (Table 2). Subtypes of other zoonotic sources, such as avian H7N9, have had mild or moderate outcomes [35]. It is worth noting that the various studies may have used different infectious doses with different viral stocks, which may lead to variability among laboratories. Focusing on specific strains within each subtype and lineage, our group and others have shown individual clinical profiles to be associated with each strain [32,85,86,87]. When analyzing the clinical outcome of seasonal virus infection, we have shown that adult ferrets infected with seasonal H1N1 virus strains A/Brisbane/59/2007 or A/Solomon Islands/03/2006 led to a mild temperature increase spiking on day 2 post-inoculation with minimal (less than 2%) associated weight loss. Moreover, after inoculation with H3N2 strains (A/Perth/16/2009, A/Brisbane/10/2007, A/Wisconsin/15/2009, and A/Victoria/210/2009), ferrets experienced a similar temperature increase but a more divergent weight loss of almost 10%, specifically after infection with A/Victoria/210/2009. In an additional study investigating a broad range of historical seasonal H1N1 strains from 1943, 1947, 1977, 1986, and 1999, we found that disease severity was generally mild, although again specific profiles were determined for antigenically divergent strains [45]. Peak temperature ranged from 101 to 104% of original temperature with no discernable hypothermic phase, and weight loss remained at less than 5% except in the case of A/USSR/90/1977 infection. In a similar study from Svitek and colleagues, the authors found ferrets infected with seasonal influenza strains also displayed strain-associated weight loss [63]. When infected, ferrets had differences in clinical signs, viral titer, lung histopathology, and immune responses specific to seasonal H1N1 or H3N2 strain [63]. Ferrets inoculated with A/PuertoRico/8/1934 experienced a peak in fever at 24 h post-inoculation, while A/USSR/90/1977-induced temperature increase was delayed and led to a peak fever after 2 days [63]. Additionally, A/PuertoRico/8/1934 caused only short-lived, mild respiratory symptoms, and had no effect on ferret body weight, while A/USSR/90/1977 caused greater weight loss, depression, and increased frequency of respiratory symptoms, similar to our findings [32]. These differences in clinical picture were apparent despite similar viral titers in nasal washes at two days post-infection. Ferrets also experienced differences in viral clearance, as A/USSR/90/1977 titers remained higher for a prolonged period of time. It is important to note that passage number and viral culture adaptations were not assessed and may have influenced disease course deviation from what would have been found for the direct human clinical isolates.

In humans, H3N2-dominant seasons are typically considered to be more severe compared to H1N1-dominant seasons [88]. Despite increased infections and hospitalizations in times of H3N2 community transmission, H3N2 infection in ferrets is generally mild (similar to seasonal H1N1 as described above), which may be an effect of strain adaptation during virus expansion in eggs [89]. Similarly, though, differential clinical pictures specific to H3N2 strains have been observed. We previously compared the H3N2 strains A/Brisbane/10/2007, A/Wisconsin/15/2009, A/Perth/16/2009, and A/Victoria/210/2009 in ferrets. While A/Wisconsin/15/2009 and A/Brisbane/10/2007 induced mild temperature increases and no discernable weight loss, A/Perth/16/2009 and A/Victoria/210/2009 led to significant fever for A/Perth/16/2009-infected animals, and almost 10% weight loss for animals infected with either strain [32]. These data suggest that ferrets may be used to determine the severity of seasonal H3N2 viruses as they emerge through clade-specific antigenic drift, although attention should be given to viral culturing prior to experimental inoculation. Similarly, in the Svitek study, individual profiles for the H3N2 strains A/Aichi/2/1968 and A/PortChalmers/1/1973 were also found, where A/Aichi/2/1968 was more severe in terms of temperature increase but led to little weight loss. Comparisons of viral load in ferrets across influenza subtypes have found that H3N2 infection led to slightly increased viral titers in the upper respiratory tract, but increased lung pathogenesis compared to seasonal H1N1 infection [32]. Lungs infected with H3N2 were marked by alveolar consolidation and pulmonary hemorrhages on day 7 post-inoculation as determined by histopathology. Other studies have found relatively mild to moderate clinical outcomes in ferrets infected with H3N2 [90,91].

### 4.2. Pandemic H1N1 Viruses in Adult Ferrets

H1N1 influenza A viruses that have gone on to cause pandemics (2009 and 1918) are typically considered to be more severe than the previously established seasonal H1N1 viruses. The 2009 H1N1 virus arose from a spillover event facilitated by previous genetic reassortment [101], while the origins of the 1918 pandemic virus are less well understood [102]. Due to the divergent nature of the epidemiology and impact of these H1N1 pandemic viruses, we considered the clinical manifestations of infection in ferrets separately, despite classification of both under the same subtype. In general, the clinical disease for pandemic H1N1 2009 infection leads to a moderate outcome, while 1918 H1N1 infection leads to increased disease severity. These are both typically more severe than seasonal H1N1 and H3N2 (discussed below) and influenza B. As the pandemic 2009 H1N1 first emerged, several groups defined the virus-induced clinical profile and transmission potential using ferrets and found moderate clinical severity, with generalized weight loss averages to be approximately 10% of original weight and a high percentage of aerosol based transmission [36,103,104]. We conducted two comparative studies, which included 2009 pandemic H1N1 influenza virus infection in ferrets [32,45]. These analyses included five different pandemic strains spanning 2009 and 2010 and revealed that, although there was strain-to-strain variability, the pandemic viruses generally induced a higher fever over several days followed by a period of hypothermia in the second half of the 14-day infection course [32,45]. Additionally, we found that the pandemic 2009 strains induced a greater amount of weight loss compared to seasonal H1N1, H3N2, or influenza B virus infection [32]. Clinical signs such as sneezing were also more apparent. In our investigations, the strain A/California/07/2009 induced the most severe clinical picture, with a greater degree of weight loss and fever compared to the other pandemic H1N1 strains. Nasal discharge and sneezing were most severe in ferrets infected with A/SouthCarolina/2/2010 and A/Mexico/4108/2009, respectively. Several studies investigating the clinical severity of the 1918 H1N1 virus have found that between 20% and 30% of original weight may be lost through the infection course, along with significant respiratory tract pathology, as noted by the number of lung lesions identified [62,84,105]. Moreover, one study by de Wit and colleagues found viral replication in the nervous system and the respiratory tract, suggesting a greater tropism of this virus and additional clinical signs involving organs outside of the respiratory tract [62]. 

### 4.3. Avian Influenza: Adult Ferrets Mirror Human Disease Severity and Are a Tool for Pandemic Preparedness

Although avian influenza viruses do not circulate globally, spillover events suggest these viruses as future pandemic threats, emphasizing the need for therapeutic and preclinical model development. Infection in ferrets with avian influenza viruses leads to a range of clinical disease signs, with the most severe (often lethal) being H5N1 infections [35,93,96,97,98,106]. Avian influenza H5N1 viruses were first characterized in ferrets in 2002 when ferrets were infected with A/HongKong/483/1997 and A/HongKong/486/97 leading to severe illness, inactivity, weight loss, prolonged temperature increases, and gastrointestinal signs in some animals [93]. In another study, ferrets infected with H5N1 (A/Vietnam/1203/04) showed 93% mortality by day 10 post-inoculation [83]. As H5N1 viruses can replicate in extrapulmonary tissues, live virus has been recovered from the upper respiratory tract, lower respiratory tract, brain, spleen, and intestines for H5N1 strains, offering an explanation for multisystem involvement during human infection with H5N1 [93,107]. Histopathological analysis of lungs from H5N1-infected ferrets revealed bronchiolitis, bronchopneumonia, and interstitial pneumonia as soon as one day post-inoculation, which only progressed over the coming days. Neurologically, the brains of infected ferrets showed immune cell infiltrates and the presence of glial nodules [93]. Additionally, H5N1 viruses assessed from 2004 outbreaks have shown higher virulence with increased lethality in ferrets, where the virus replicated to higher titers [96]. This finding has only increased the concern over H5N1 strains as spillover threats. A commonality identified for the H5N1 strains from these studies is the neurotropic distribution and multiorgan infection outside of the respiratory tract [96]. Comparatively, other avian-derived strains, including the strain of concern H7N9 which has caused significant human outbreaks in China with a case-fatality rate between 20% and 30%, have led to only mild disease in ferrets [35,106]. In a study from Zhu and colleagues, ferrets infected with human H7N9 virus isolates had observable fever, but lost less than 5% of original weight and had no other observable clinical signs [35]. Another study comparing the H7 subtypes H7N7 and H7N2 also found moderate weight loss for all except the H7N7 strain A/Netherlands/219/2003 [98]. Minimal clinical disease has been observed for H6 influenza viruses inoculated in ferrets [97].

### 4.4. Ferrets Suggest Influenza B Viruses Should Not Be Ignored

Type B influenza viruses seasonally circulate across the globe, contributing to influenza virus-associated morbidity and mortality, particularly among children and older individuals [108]. Ferrets are susceptible to influenza B viruses of both Yamagata and Victoria lineages allowing antigenic, clinical, and immunological characterization of these viruses as they undergo antigenic drift [8,32,109,110]. Following inoculation with influenza B viruses, ferrets typically develop a rise in temperature peaking at two days post-inoculation and display nasal discharge. Weight loss and sneezing may occur following inoculation with some strains [8], indicating strain-specific clinical disease [8,32]. In a study of influenza B virus infection in ferrets by our group, disease severity was not attributable to a particular lineage, but rather depended upon the strain [8]. In particular, although all ferrets were infected with the same infectious dose of influenza B viruses, the B Victoria lineage strain B/Brisbane/60/2008 elicited the most pathology and even led to lower respiratory tract infection compared to B/Bolivia/1526/2010 and Yamagata strains B/Florida/04/2006 and B/Wisconsin/01/2010. Although influenza B virus epidemics have been severe in humans, lower respiratory tract infection is typically not associated with influenza B viruses, and tropism is considered to be restricted to the upper respiratory tract. These data suggest that some influenza B viruses can induce greater pathogenesis with lower respiratory tract infection, accounting for public health records of severe disease and mortality, even in healthy adult people.

### 4.5. Immune Response Dissection Following Influenza Virus Infection in Adult Ferrets: Identification of Immune Correlates and Pathogenic Mechanisms

Now that we identified the clinical disease characteristics associated with specific influenza viral infections in adult ferrets, we will now discuss immune markers of severity correlating with clinical disease. Studies by others and us have shown that the ferret cytokine response mirrors that of humans after infection with seasonal, pandemic, and avian influenza viruses, which is strain-specific. 

In one study, investigators compared the cytokine profiles after infection with influenza viruses strains that led to moderate or mild disease. In the case of mild disease from A/Aichi/2/68 or A/Puerto Rico/8/34 infection, ferrets showed strong upregulation of IFN-alpha, IFN-gamma, and TNF-alpha early after infection, while the more pathogenic strains investigated, A/USSR/90/1977 and A/Port Chalmers/1/73, elicited delayed activation of IFN-alpha and IFN-gamma [63]. Although these cytokines were both associated with mild and moderate influenza in this study, the timing of cytokine induction may be an important factor for marking disease severity. Additionally, IL-8 was induced after mild infection, while elevated IL-6 was associated with severe influenza virus strains [63].

After the 2009 pandemic H1N1 emerged, it was suggested that the new pandemic virus was more pathogenic than the previous seasonal H1N1 viruses. To investigate the markers of disease severity associated with the pandemic H1N1 virus, Rowe et al. used the ferret model to compare the host responses to the seasonal H1N1 virus A/Brisbane/59/2007 [92]. Key chemokines CCL2, CCL8, CCL13, CCL19, CXCL7, and CXCL10, and interferon stimulated genes including IRF1, IRF2, SOCS1, STAT1, STAT2, ISG15, and ISG20 were shown in response to pandemic H1N1, A/California/07/2009. This was accompanied by pathology in the lungs that was significantly increased in response to A/California/07/2009 compared to A/Brisbane/59/2007. In response to pandemic H1N1, ferret lungs had severe alveolar pneumonia, moderate edema, and infiltration of inflammatory cells. As early as day 1 post-infection, infiltrating leukocytes were associated with damage to vascular walls and bronchial epithelial necrosis was observed. Damage in the lung diminished once adaptive immune genes were upregulated, which corresponded to detectable antibodies and viral clearance. It was suggested that a poor switch to the adaptive immune system, as demonstrated in the ferret model, could lead to poor outcomes in humans. 

In a similar follow-up study comparing the immune responses of seasonal H1N1 virus infection to pandemic 2009 H1N1 by use of sequencing technology, the inflammatory markers IL-6, IFN-alpha, and TNF-alpha were significantly increased in the pandemic 2009 H1N1-infected ferret lungs [111]. In this large-scale immune analysis by transcriptome profiles, the ferret infectome indicating the immune signatures induced after pandemic 2009 influenza virus infection in the respiratory tract and lymph nodes were defined [50]. On day 5 post-inoculation, 2926 genes were significantly upregulated and 637 genes downregulated in the lung, characterized by increased interferon-stimulated genes (CXCL10, OAS1, and IRF1) and increased levels of proinflammatory and immunomodulatory cytokines and chemokines (IL-1beta, IL-6, IL-8, IL-27, CXCL11, CXCL16, CCL3, CCL4, and CCL5). Further immune analysis suggested the peaking of innate immune responses in the lung on day 3 post-inoculation, followed by a transition to an adaptive profile between days 5 and 7. Conversely, the immune profile from the lymph node was skewed toward downregulation of immune genes (day 5 post-inoculation), where upregulated genes were enriched by interferon-stimulated genes. This gene signature may represent the classical immune response, which is initiated after antigen is carried to the draining lymph node.

The 1918 H1N1 and H5N1 are regarded to be more highly pathogenic than typical seasonal influenza viruses. Therefore, identifying immune drivers of pathogenesis is a research priority. In a study investigating the immune drivers of the 1918 H1N1 virus severity, the proinflammatory cytokines IL-6, IL-8, and TNF-alpha were investigated since these cytokines have been previously associated with inflammation. These cytokines are not only increased in the respiratory tract, but also in the central nervous system, liver, heart, and pancreas, providing immunopathological mechanisms accounting for disease severity [62]. In an H5N1 study comparing a lethal, highly pathogenic avian influenza H5N1 strain (A/Vietnam/1203/2004) to ferrets infected with a milder H3N2 strain (A/Panama/2007/1999), a functional genomic analysis found enrichment of interferon response genes, with a focus on the CXCL10 pathway [39]. These data suggested a pathologic role for CXCL10 and its chemokine receptor CXCR3 in the disease severity induced by H5N1. This was further interrogated by a CXCR3 antagonist, AMG487, which validated the contribution of this pathway to H5N1 pathogenesis and increased severity of the H5N1 subtype compared to the seasonal H3N2 influenza viruses [39].

After review of the above published studies, trends have emerged in regard to the cytokine regulation of more pathogenic viruses. Specifically, the inflammatory markers IL-6 and CXCL10 have been upregulated in the models of more severe disease. While identification of this trend is important, it is prudent to keep in mind that not all of the reviewed studies took a comprehensive approach to determining the immune responses and disease severity marker discovery. Additional work is needed, which may suggest specific mechanisms leading to disease for each virus.

## 5. Studying Influenza in the Extreme Aged with the Aged Ferret

### 5.1. Disease Severity Increases with Ferret Age

Older individuals have the greatest mortality risk associated with influenza [2]. Due to the impact of older age on influenza severity, it is essential to develop a model for investigating the contribution of older age, including the aspects of immunosenescence and inflamma-ageing on influenza disease severity. Older ferrets display features of aging and comorbidities similar to those of humans including hair loss, increased incidence in tumors and neoplasia, arthritis, cataracts, cardiomyopathy, kidney disease, and gastrointestinal issues [112]. They are considered old past 2 years of age, but may live as long as 10 years as a companion animal. Despite the ability of older ferrets to serve as a model, relatively few studies have employed aged ferrets to investigate influenza virus infection and the immunopathogenic mechanisms of disease. 

In one study, aged ferrets (5.5–7 years) infected with 2009-pandemic H1N1 virus A/California/07/2009 experienced more severe disease as identified by more pronounced weight loss with increased mortality compared to infected adult ferrets, aged 6–12 months [25]. Significant pathology was noted in the respiratory tract of adult and aged animals. Both bronchial and alveolar pneumonia was observed in both age groups. In the adult, pneumonia peaked at day 5 and was ultimately cleared by day 8, while aged animals showed peak pneumonia on day 8. In addition, the peak percentage of lung involvement in terms of severe pathology was also on day 8 when euthanasia was required for aged animals. Aged ferrets showed minimal bronchial involvement in pathology, which steadily increased throughout the time course. When examining the degree and location of infecting virus, early on aged animals showed minimal evidence of infection, with the alveolar compartment being most affected. Virus in the bronchioles peaked on day 5 in the aged, as compared to days 1 through 5 in adult animals. The virus was unable to be cleared by day 8. In addition, aged animals showed significantly worse infection in both the trachea and the submucosal gland when compared to adult animals [25]. Chen et al. examined the role of glycosylation in the progression of influenza virus-induced disease in an aged ferret model [77]. In their study of the 2009-pandemic H1N1 (A/California/07/2009) infection in aged ferrets, the authors found that glycosylation levels vary significantly with age. The lungs of aged animals showed higher levels of α-2,6-sialosides, one of the receptors for 2009-pandemic H1N1 influenza viruses, and loss of O-linked α-2,3-sialosides, which may play a role in viral clearance and disease progression. A correlation with mannose levels was noted when observing trends in pathology between adult (6 months to 1 year) and aged animals (5.5–7 years). While adult animals developed pathology early that was ultimately resolved, they also had mannose levels peak on day 1 post-infection. In contrast, aged animals developed pathology later in the infection course that did not resolve. The lungs of aged animals also showed high levels of mannose on day 3 that remained until day 8 post-infection [77]. As with the human population, the elderly are more at risk for severe outcomes resulting from influenza virus infection. The ferret model continues to be an important tool to discover mechanisms related to this discrepancy in disease progression between the adult and aged populations.

### 5.2. Influenza Disease Severity Is a Function of Age and Previous Infection

As we discussed here, influenza disease severity increases with age. With this understanding, we recognized that as we age, we accumulated influenza virus exposures, leading to infections and recovery and facilitating immune memory establishment. Therefore, clinical outcomes in older individuals may be influenced by both age-related immune declines, and pre-existing immunity acquired from previous infections. In short, influenza disease severity is the intersection of previous infection and age. Determining how the host factors of age and previous infection influence disease is a significant research priority for the influenza field. To address this knowledge gap, we employed the aged ferret model in an infection–reinfection study in an attempt to better represent the immune status and influenza disease of this age group [78]. Aged ferrets greater than 4 years of age were infected sequentially with homologue viruses, two 2009-H1N1 pandemic viruses (A/Mexico/4108/2009 followed by A/California/07/2009), or heterologous H1N1 viruses (A/Brisbane/59/2007 followed by A/Mexico/4108/2009), first being infected with a previously circulating seasonal H1N1 from 2007 and then re-infected with an antigenically distinct 2009-H1N1 pandemic virus. While aged animals showed comparable clinical outcomes during homologous infection, weight loss was significantly greater than the weight loss of adult ferrets (aged 4–6 months) during heterologous infection, despite comparable viral titers (10^2.5^ TCID_50_/mL) [53]. During heterologous infection, antibodies elicited against the first virus were delayed until day 14, compared the adult ferrets who had specific antibodies present on day 7 [53]. In agreement with the HI data, the B cell marker CD19 was decreased in aged ferrets during heterologous infection [53]. Blood analysis of T cell markers by qRT-PCR revealed that while prominent T cell responses were evident in adult animals, these responses were significantly blunted in aged animals, with notable decreases in CD4, CD8, and the T cell activation marker CD28. Furthermore, markers of the Th1 response were examined, given their prominent role in heterologous immunity. The Th1 transcription factor TBX21, and type 1 cytokines IFN-gamma and TNF-alpha, were all significantly reduced in aged ferrets compared to those in adults [53]. Together, this implied a potential age-related decrease in lymphocyte responses, particularly Th1 responses, which may indicate T cell exhaustion that could contribute to more severe disease in the aged population. 

### 5.3. Another Stage of Life: Pregnancy

Although not a traditional age group, pregnancy represents a unique physiological state that can impact infection outcomes in mother and fetus. Influenza virus infection in a pregnant host was first modeled in ferrets in the 1970s. Early work by groups including Sweet et al. have demonstrated the unique infection outcomes that accompany a viral infection during pregnancy using pregnant ferrets. In their 1977 study, the researchers inoculated pregnant ferrets at early, middle, and late gestation (12–20, 21–29, and 30–42 days post coitus, respectively) using A/PR/8-A/England/939/69 clone 7a. Virological analysis suggested that tissues unique to pregnancy could support viral replication, including the placental barrier and amniotic fluid [29]. Importantly, infection of the fetus itself was not possible when the mother was intranasally infected, but only when intracardially infected at high viral titer, which does not naturally occur, suggesting little risk of direct inoculation to the fetus during seasonal respiratory infection [29]. Additionally, intracardial infection of the mother only led to infection when inoculated during late stages of gestation [29]. Infection at later gestational stages had an effect on pregnancy outcomes, with the infected pregnant ferrets delivering significantly fewer kits than their uninfected counterparts. This work led to a follow-up study published in 1983, which showed that intracardial influenza A infection (A/PR/8-A/England/939/69 clone 7a) in pregnant ferrets led to lesions in the respiratory tract and liver of the fetus. Lesions first appeared in the nasal turbinate before appearing in the upper respiratory tract, suggesting that infection in the fetus occurred through the amniotic fluid and not through the blood stream [31]. Additional studies with A/PR/8-A/England/939/69 clone 7a have been performed using intra-amniotic inoculation to infect a fetus with influenza virus during the early period of gestation [76]. The majority of these infections did not lead to viable offspring. Few pregnant mothers gave birth, with most fetuses being absorbed at late gestation approximately 19-21 days post-inoculation. A few mothers did reach full term and delivery that resulted in stillbirths [76]. These infections also led to lesions in the reproductive organs of the pregnant mothers [76].

In addition to the outcomes of the fetus, the progression of disease in the pregnant female herself is of concern, since pregnancy is considered to cause an immunocompromised state [21,75]. It was determined early in the development of this model that intranasal infection with A/PR/8-A/England/939/69 clone 7a in a pregnant animal could lead to significantly higher fever than in a non-pregnant animal [113]. More recent studies by Yoon et al. investigating the effect of the 2009-pandemic H1N1 on pregnant females utilized the ferret model [75]. In these studies, both pregnant and non-pregnant animals were intranasally inoculated with A/California/07/2009. While effects on the fetus appeared minimal, infected pregnant mothers demonstrated increased proinflammatory cytokines, including IFN- α, IL-4, TNF- α, IL-6, and IFN- γ, and decreased IL-10, throughout the respiratory tract. In addition, lower circulating B cell and CD8+ T cell numbers were found in the blood of pregnant animals compared to their non-pregnant counterparts [75].

## 6. Ferret Age Models in Vaccine Research

Given that ferrets are incredibly versatile models of influenza virus infection, they are valuable models for testing influenza vaccines and therapeutics. Many newly developed influenza vaccines have been evaluated in ferrets in addition to being evaluated in mice. The vaccines investigated in the ferret model have included a wide variety of different platforms. For example, emerging influenza viruses have stimulated research into vaccines against emerging avian influenza viruses H5N1 [114,115], H7N9 [116,117,118,119,120,121], and the pandemic 2009 H1N1 influenza virus [122,123,124,125]. This has included platforms such as live-attenuated influenza strains [114,120], inactivated vaccines [115,116], viral vectors [119], VLPs (virus-like particles) [118], and mRNA vaccines [121]. Although many have been protective in both mouse and ferret models, none have yet gained approval for general use.

The evaluation of seasonal influenza vaccines in ferret models has also become more common. For example, a recent seasonal vaccine was updated to a quadrivalent version and was demonstrated in ferrets to have superior protection against influenza B over the trivalent version [126]. Many studies have also developed new platforms in an effort to create a universal vaccine that can protect against all seasonal strains without yearly reformulation. The majority is based on either live attenuated [124,127,128] or non-live [129,130,131,132,133] approaches. While many of them have been protective, only one has advanced into clinical trials at this time [124].

Despite these many advancements in influenza vaccinology, the vast majority of vaccines evaluated in ferrets have been designed for the human adult population and have been tested in healthy, adult ferrets. With the development of the infant, pregnancy, and aged ferret models that have been described in this review, it is important to develop a strategy for the utility of these models for age-specific vaccine development, especially considering the high-risk of the extreme age groups. For example, the comparative efficacy of the high-dose seasonal vaccine developed by Sanofi for those >65 years of age could be assessed in aged ferrets. Similarly, it would be valuable to test novel adjuvants such as the surfactant-conjugated cGAMP described by Wang et al. in the aged ferret model [134]. The safety and efficacy of live and non-live vaccines could be assessed in the infant and pregnant models, and the impact of influenza infections on developing fetuses could also be assessed in the pregnant model. It is well known that infants have a lower immune stimulation leading to decreased protection from influenza vaccination. The infant ferret models described could also be used to evaluate adjuvants for this age group such as MF59, which is used in FLUAD pediatric [14].

## 7. Conclusions

The ferret is a small preclinical research model which has been identified as susceptible to human respiratory viruses. In humans, respiratory viruses cause a spectrum of disease severity, which is often correlated with age. Importantly, this phenomenon is not unique to circulating viruses but also is an outcome of infection with novel viruses recently spilled over from animal reservoirs such as the 2009 H1N1 pandemic influenza virus and now for severe acute respiratory syndrome coronavirus 2 (SARS-CoV-2). Understanding how age affects respiratory virus disease is essential for developing the most effective countermeasures, which may be host- and virus-specific. Here we discussed how ferrets are able to recapitulate age-related influenza disease. We recognize that additional work is needed to fully elucidate the disease mechanisms of age-related illness for which the age ferret models may be of use. Furthermore, as we have highlighted, the use of age-specific influenza virus infection may be utilized for age-specific vaccine development.

## Figures and Tables

**Table 1:** Limited ferret-specific reagents are available for immune response and pathogenesis dissection following influenza virus infection or vaccination (modified from Albrecht et al. [38]).

Commercial Kits			
Application	Product Type and Name	Vendor	Refs
**Flow cytometry**	LIVE/DEAD Fixable Aqua dead cell stain	Thermo/Fisher	[54]
**ELISpot**	IFN-gamma ELISpot basic (HRP) kit	MabTech	[54]
**Primary Antibodies**			
**Application**	**Product Type and Name**	**Vendor**	**Refs**
**Flow cytometry**	CD44	BD Pharmingen	[55]
	IL-4	Bio-Rad	[55]
	IFN-gamma	Bio-Rad	[55]
	IFN-gamma	BD Pharmingen	[55]
	TNF	BD Pharmingen	[55]
	Thy1.1	BD Pharmingen	[55]
	CD11b	BD Pharmingen or BioLegend	[54,55]
	CD8a	eBioscience/Tonbo	[54,55]
	CD4	Sino Biological	[54,55]
	MHC-II	BioLegend	[54]
	IgA, IgM, IgG	LSBio	[54]
	CD59	BD Pharmingen	[54]
	CD79a	eBioscience	[54]
	CD20	Sino Biological	[54]
	CD3	Santa Cruz Biotech	[56]
**Flow cytometry/ELISpot**	IFN-gamma (capture Ab)	Bio-Rad	[56]
	IFN-gamma biotinylated (detection Ab)	R&D Systems	[56,57]
**Immuno-histochemistry**	CD3	Dako	[58]
	Lysozyme	Dako	[58]
	CD20	Thermo/Fisher	[58]
	CD79a	Dako	[58]
	MHC-II	Dako	[58]
**Real-Time PCR Primers**			
**Application**	**Product Type and Name**	**GenBank or NCBI Accession No.**	**Refs**
**Housekeeping genes**	Beta-actin	AF038150 and NM_007393.3	[24,50,59,60]
**Interleukins**	IL1-2p40		[61]
	IL-2		[61]
	IL-4		[61]
	IL-6	NM_031168.1	[60,61,62]
	IL-6ra	NM_010559.2	[60]
	IL-8		[62,63]
	IL-10		[1,61]
**Interferons and ISGs**	IFN-alpha		[61]
	IFN-gamma		[61]
	OASL		[23]
**Chemokines**	CCL2		[24]
	CCL5	MPF80001524	[50]
	CXCL9		[23]
	CXCL10	EF492058	[23,24,50,59]
	CXCL13		[23]
	CCL19		[23,24]
	CCL21		[23]
**Immune cell receptors**	CD3e		[23]
	CD8a	EF492056	[50]
	CD11c		[23]
	CD19		[23]
	CD79a	MPF80001635	[50]
	CD80	MPF80001637	[50]
	CD86	MPF80001642	[50]
**Immunoglobulin and MHC genes**	IGHG	GD183042	[50]
	IGHM	GD183075	[50]
	MHC-I		[23]
	MHC-II		[23]
**Other genes**	CSN2		[24]
	FOS		[24]
	GAPDH		[61]
	LPL		[24]
	MSR1		[24]
	ORM2	NM_011016.2	[60]
	SAA3	NM_011315.3	[60]
	SAA4	NM_011316.3	[60]
	SOCS1	NM_009896.2	[60]
	SOCS3	NM_007707.3	[60]
	STAT3	NM_213659.2	[60]
	STAT5a, STAT5b		[24]
	TNF-alpha		[61,62,63]
	TGF-beta1		[23]

**Table 2:** Infection with influenza virus types A and B show variable, strain-dependent clinical pictures in ferrets.

Influenza A (Seasonal)								
		Clinical Parameters						
Subtype	Strain	Fever ^a^	Weight Loss ^b^	Nasal Secretions ^c^	Sneezing ^c^	Neurotropism (Yes/No) *	Other	Refs
**H1N1**	A/AA/Marton/1943	++	+	+++	+	NI		[45]
	A/FortMonmouth/1/1947	+	+	+	+	NI		[45]
	A/USSR/90/1977	+	++	+++	+	NI		[45,63]
	A/Taiwan/1/1986	+	+	+++	+	NI		[45]
	A/NewCaledonia/20/1999	++	++	+++	+	NI		[45]
	A/SolomonIslands/03/2006	+	+	++	+	NI		[32]
	A/Brisbane/59/2007	+	+	+	+	NI		[32,92]
	A/NewYork/18/2009	++	++	++	++	NI		[45]
**H3N2**	A/PortChalmers/1/73	+	+	+	+	NI	Depression	[63]
	A/Sydney/05/1997	+	NI	NI	NI	Yes		[93]
	A/Panama/2007/1999	+	NI	+++	+	Yes		[39,93]
	A/Brisbane/10/2007	++	++	+++	++	NI		[32]
	A/Perth/16/2009	+++	++	++	++	NI		[32]
	A/Wisconsin/15/2009	+	+	+	+	NI		[32]
	A/Victoria/210/2009	++	++	+	+	NI		[32]
**Influenza A (Pandemic)**								
		**Clinical Parameters**						
**Subtype**	**Strain**	**Fever ^a^**	**Weight Loss ^b^**	**Nasal Secretions ^c^**	**Sneezing ^c^**	Neurotropism (Yes/No) *	**Other**	**Refs**
**H1N1**	1918 H1N1 virus	NI	++	++	+++	Yes		[62]
	A/California/07/2009	++	++	++	+	NI		[32,50,92]
	A/Mexico/4108/2009	++	++	++	++	NI		[32,50]
	A/Utah/20/2009	++	+	++	+	NI		[32]
	A/SouthCarolina/2/2010	+++	++	+++	+	NI		[32]
**Influenza A (Avian)**								
		**Clinical Parameters**						
**Subtype**	**Strain**	**Fever ^a^**	**Weight Loss ^b^**	**Nasal Secretions ^c^**	**Sneezing ^c^**	Neurotropism (Yes/No) *	**Other**	**Refs**
**H5N1**	A/HongKong/483/1997	+++	+++	++	+	Yes	Diarrhea in some animals	[93]
	A/HongKong/486/1997	+++	++	+	++	Yes	Mucopurulent nasal discharge, diarrhea in some animals	[93]
	A/Vietnam/1203/04	+++	+++	++	++	Yes	Diarrhea, mortality in some animals	[39,83,94,95,96]
**H6NX**	A/teal/HK/W312/1997 A/quail/HK/1721-30/1999 A/mallard/Alberta/89/1985 A/duck/HK/182/1977	+	+	+	+	NI		[97]
**H7N2**	A/Tky/VA/4529/1902	+	++	++	++	Yes		[98]
	A/NY/107/2003	+	++	++	++	Yes		[98]
**H7N7**	A/Netherlands/230/2003	++	++	+	+	Yes		[98]
	A/Netherlands/219/2003	++	+++	+++	+++	Yes	Diarrhea in some animals	[98]
**H7N9**	A/Shanghai/2/2013	+	+	+++	+++	Yes		[35,99]
	A/Anhui/1/2013	++	++	NI	NI	Yes	Lethargy, inappetence, breathing difficulties	[99,100]
**Influenza B**								
		**Clinical Parameters**						
**Lineage**	**Strain**	**Fever ^a^**	**Weight Loss ^b^**	**Nasal Secretions ^c^**	**Sneezing ^c^**	Neurotropism (Yes/No) *	**Other**	**Refs**
**Yamagata**	B/Florida/04/2006	++	+	+++	+	NI		[8,32]
	B/Hubei-wujiagang/158/2009	++	+	+	+	NI		[32]
	B/Wisconsin/01/2010	+	+	++	+	NI		[8]
**Victoria**	B/Brisbane/60/2008	++	++	+++	+	NI	Lower respiratory tract infection	[8,32]
	B/Bolivia/1526/2010	++	+	++	+	NI		[8]

^a^ Fever: mild (+) = increase <2% for a single day; severe (+++) = 40 °C or 103% baseline for >1 day; ^b^ Weight loss: mild (+) = <5% lost; severe (+++) = >15% lost; ^c^ Respiratory symptoms: mild (+) = observed in <30% animals; severe (+++) = observed in >60% animals; * NI = not investigated.

## Data Availability

Not applicable.
